# Blood Particulate Analogue Fluids: A Review

**DOI:** 10.3390/ma14092451

**Published:** 2021-05-09

**Authors:** Samir Hassan Sadek, Manuel Rubio, Rui Lima, Emilio José Vega

**Affiliations:** 1 Departamento de Ingeniería Mecánica, Energética y de los Materiales and Instituto de Computación Científica Avanzada (ICCAEx), Universidad de Extremadura, E-06006 Badajoz, Spain; sadek@unex.es (S.H.S.); marubio@unex.es (M.R.); 2 MEtRICs, Mechanical Engineering Department, Campus de Azurém, University of Minho, 4800-058 Guimarães, Portugal; rl@dem.uminho.pt; 3 Transport Phenomena Research Center, Department of Chemical Engineering, Faculty of Engineering, University of Porto, Rua Dr. Roberto Frias, 4200-465 Porto, Portugal

**Keywords:** blood analogue, microparticle, RBC templates, biomicrofluidics

## Abstract

Microfluidics has proven to be an extraordinary working platform to mimic and study blood flow phenomena and the dynamics of components of the human microcirculatory system. However, the use of real blood increases the complexity to perform these kinds of in vitro blood experiments due to diverse problems such as coagulation, sample storage, and handling problems. For this reason, interest in the development of fluids with rheological properties similar to those of real blood has grown over the last years. The inclusion of microparticles in blood analogue fluids is essential to reproduce multiphase effects taking place in a microcirculatory system, such as the cell-free layer (CFL) and Fähraeus–Lindqvist effect. In this review, we summarize the progress made in the last twenty years. Size, shape, mechanical properties, and even biological functionalities of microparticles produced/used to mimic red blood cells (RBCs) are critically exposed and analyzed. The methods developed to fabricate these RBC templates are also shown. The dynamic flow/rheology of blood particulate analogue fluids proposed in the literature (with different particle concentrations, in most of the cases, relatively low) is shown and discussed in-depth. Although there have been many advances, the development of a reliable blood particulate analogue fluid, with around 45% by volume of microparticles, continues to be a big challenge.

## 1. Introduction

The study of the blood flow behaviour through microchannels is crucial to improve our understanding of phenomena happening in the human microcirculatory system. The development of microfluidics technology has allowed the production of platforms that replicate the microvascular system with applications to detect and study pathologies (diseases), to assess drug treatments, among others [1,2,3], providing many new insights into the physical, chemical, and physicochemical responses of cells [4]. However, there are difficulties associated with the use of in vitro blood, such as coagulation, sample storage, sample disposal, complex cleaning of microdevices used, etc., in addition to ethical and economic issues. These drawbacks have promoted the increasing interest to develop fluids with rheological properties similar to real blood [5]. The first and most conventional blood analogues used in experimental flow studies are Newtonian fluids using a mixture of water/glycerol and water/DMSO (dimethyl sulfoxide). These are the simplest blood analogue fluids to be produced and have been applied in a wide range of biomedical applications from large arterial models to microfluidic devices [6,7,8,9,10,11,12,13,14,15]. Other popular blood analogues are the non-Newtonian fluids where additives including xanthan gum (XG) and/or polyacrylamide (PAA), sodium iodide, and urea are often diluted in glycerol and/or water and have also been applied in different kinds of flow studies [5,10,16,17,18,19,20,21]. However, blood analogue liquid solutions that only take into account the rheological behaviour are not enough to ensure an accurate representation of several blood physiological phenomena happening in microcirculation.

Blood is not exclusively liquid, it is a complex physiological dispersion composed of plasma (around 55 vol%), red blood cells (RBCs, around 45 vol%), and white blood cells/platelets (around 1 vol%) [22]. As RBCs are the most abundant particles in the blood, they manage the behaviour of the blood flow through microvessels (microchannels). Agglomeration, disaggregation, and deformations of RBCs explain the non-Newtonian shear-thinning behaviour exhibited by blood [16,23,24]. When blood flows through vessels, with diameters less than 300 microns, the apparent blood viscosity scales with blood vessel diameter due to lateral migration of RBCs away from the vessel wall towards the center of the current, the so-called Fähraeus-Lindqvist effect. The lateral migration of RBCs produces the formation of a cell-free layer (CFL) next to the vessel wall [1,23], i.e., hematocrit reduction and increase in the walls and center, respectively. In order to reproduce multiphase effects mentioned above, the blood analogue solution should contain a large volume of particles (around 45% by volume) that mimic key structural attributes of RBCs, including size, shape, and mechanical properties. The development of more reliable blood analogues with particles is of great importance and interest for the development of precise studies in vitro of Bioengineering, in general, and Biomicrofluidics, in particular, aimed at obtaining knowledge to improve the quality of Public Health.

It should be pointed out that blood analogue fluids called “blood mimicking fluids” (BMFs) began to burst in at the end of the 20th century, which are nowadays commercialized [25,26]. These fluids, in most cases, have dispersed rigid microparticles inside (polystyrene, nylon, etc.). In general, these BMFs have been developed to comply, as faithfully as possible, with physical (density, viscosity, and particle size) and acoustic (speed, backscatter, and attenuation) properties for their specific use in the improvement and calibration of Doppler ultrasound. BMFs are also used for the evaluation of magnetic resonance techniques, or to perform particle image velocimetry (PIV). Therefore, BMFs are out of the scope of this manuscript. Anywise, as will be seen, the stiffness of the microparticles used in these commercial fluids makes them unsuitable for the purpose presented here.

In this review, the evolution over the last two decades to produce a reliable blood particulate analogue fluid is revised and discussed. The paper is organized as follows. The morphology, mechanical properties, and even biological functionalities of the microparticles produced and used to mimic RBCs, besides the methods developed to fabricate these RBC templates, are presented and analyzed in Section 2. In Section 3, the dynamic flow/rheology of blood particulate analogue fluids proposed in the literature (with different particle concentrations) is shown and discussed, including shear and extensional rheology, and studies about the CFL. The paper closes with the main conclusions in Section 4, including perspectives and future issues.

## 2. RBC Templates

Normal red blood cells (RBCs), or erythrocytes, have a biconcave discoidal morphology and contain hemoglobin, offering extraordinary biological functionalities: great flexibility to deform and squeeze through microcapillary vessels, even with strict restrictions, recovering their shape after crossing them; the ability to circulate long-term in human body (up to 120 days); and the ability to deliver oxygen ($O_{2}$) to the body tissues (taking advantage of the large surface area-to-volume ratio for rapid gas exchange). A typical human red blood cell has a disk diameter of approximately 8 μm and a thickness at the thickest point of around 2 μm and a minimum thickness in the center of around 1 μm [27,28]. An ideal RBC template should be able to mimic the structural attributes of a real RBC: size, shape, and mechanical properties, besides its biological functionalities. Although the high throughput production of RBC templates that fulfill all these requirements continues to be a big challenge, in recent years, there have been important advances. In this section, it should be pointed out that we will focus our attention on particles with at least a similar size to that of RBCs, despite the fact that a few works can be found in the literature fabricating/using particles with sizes smaller than 5 μm [29,30,31,32].

### 2.1. Production Methods, Size, and Shape

**Figure 1:**
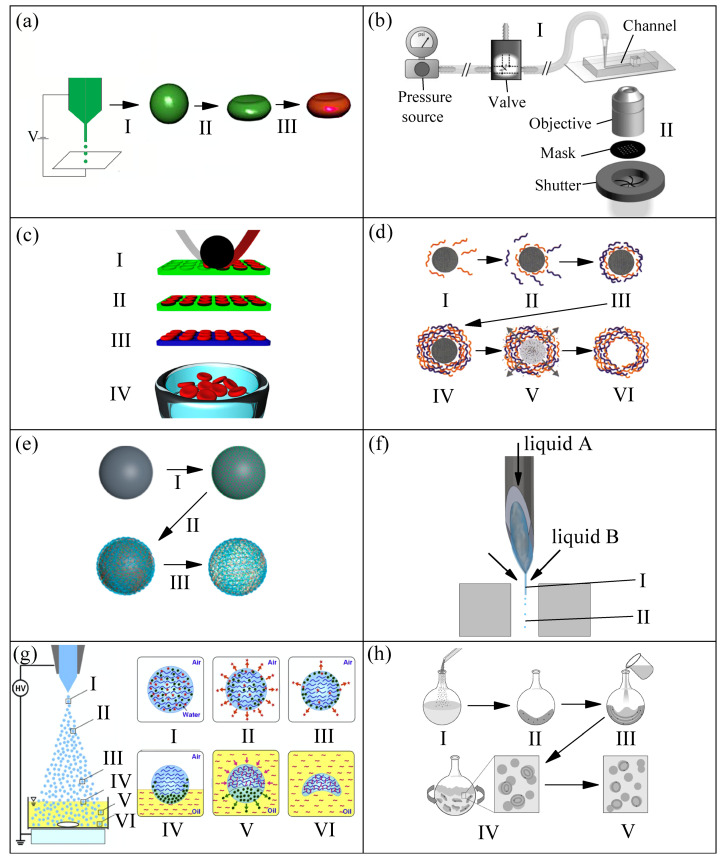

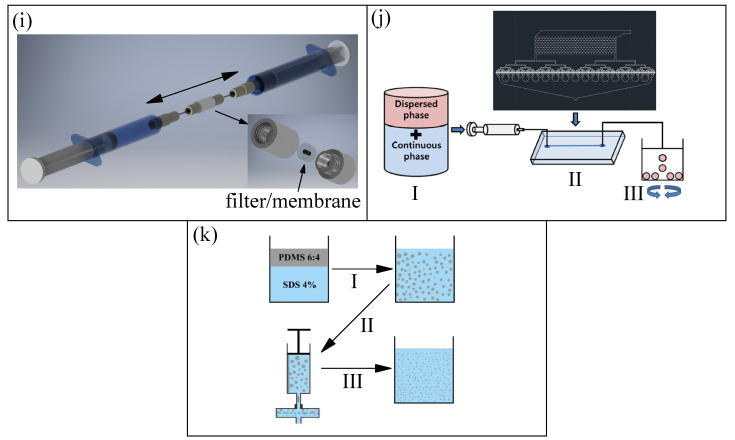
Production methods of RBC templates: (**a**) electrohydrodynamic jetting [33,34], (**b**) stop flow lithography (SFL) [35,36], (**c**) PRINT^®^ [37,38], (**d**) Layer-by-Layer [40,41,42], (**e**) $CAP_{ATRP}$ [43], (**f**) liquid–liquid flow-focusing with a hypodermic needle [44,45,46], (**g**) electrospray with solvent diffusion [47], (**h**) lipid film hydration [48,49], (**i**) two-syringe membrane emulsification [50], (**j**) 512-channel geometric droplet-splitting microfluidic device combined with a post array part [51], and (**k**) premix membrane emulsification [52]. Roman numerals are used to explain, in the text, the main steps for each production method. Reproduced from Refs. [34,35,37,40,43,44,47,48,50,51,52] with permission from National Academy of Sciences, WILEY-VCH Verlag GmbH, American Chemical Society, AIP Publishing, Elsevier Science INC, MDPI, and The Royal Society of Chemistry.

During the last twenty years, the development of methods to produce microentities to mimic the particulate nature of blood has become a popular research topic due to the advantages that blood particulate analogue fluids may offer over conventional ones. Figure 1 show and summarize the methods developed for the production of RBC templates, following a chronology order. Figure 1a electrohydrodynamic jetting [33,34]: (I) production of poly(lactic acid-co-glycolide) (PLGA) microspheres by electrospray, a biocompatible and biodegradable polymer, (II) incubation in 2-propanol for obtaining PLGA RBC-shaped template, and (III) Layer-by-Layer (LbL) coating on template, protein cross-linking, and dissolution of template core yielded the final biocompatible RBC templates, 7 ± 2 μm in size. Figure 1b stop flow lithography (SFL) [35,36]: polyethylene glycol (PEG) hydrogel particles are formed in a stationary layer of monomer sandwiched inside a PDMS microchannel before being flushed out, the process is repeated cyclically. The monomer solution consisted of 65% *v*/*v* PEG mixture containing PEG diacrylate with a molecular weight of 700 and PEG with a molecular weight of 200, 15% *v*/*v* photoinitiator 2-Hydroxy-2-methyl-1-phenylpropan-1-one, and 20% *v*/*v* TE buffer. (I) the pressure-driven flow of monomer solution through the device is stopped, (II) an array of portions of particles is polymerized (crosslinked) using mask-defined UV light by opening the shutter briefly, and (III) the solidified discoidal particles, 8 ± 0.2 μm in diameter, are flushed out at high velocity. Figure 1c particle replication in nonwetting templates (PRINT^®^) [37,38]: (I) hydrogels composed primarily of 2-hydroxyethyl acrylate (HEA) were used that were lightly (1–10%, by weight) cross-linked with poly(ethylene glycol) diacrylate (PEGDA) with a photoinitiator (1-hydroxycyclohexyl phenyl ketone), and polymerizable fluorescent dyes (1%) to facilitate imaging (to mimic the negatively charged RBC membrane, it was also added 2-carboxyethyl acrylate), disc-shaped gaps of elastomeric fluoropolymer mold (green) were filled by the prepolymer mixture (red) using a roller (black) covered by a high-surface-energy sheet (gray) to wick away excess liquid, (II) the filled mold was cured photochemically under UV light, yielding cross-linked hydrogel particles, (III) the particles were harvested from the mold by freezing onto a thin film of 1% poly(vinyl alcohol) in water (blue), removing the mold, (IV) and a suspension of hydrogel particles, around 6 μm in size, were obtained after the melting of that thin film. One year later, this method (PRINT^®^) [38] was also used to fabricate microparticles of triethylene glycol acrylate (TEGA) and 2-carboxylethyl acrylate (CEA, 10% by weight), using a very low amount of cross-linker (PEGDA), and with the incorporation of bovine hemoglobin (Hb) into RBC templates to enhance oxygen transport. A electrohydrodynamic technique (electrospraying), similar to that depicted in Figure 1a-step( 1), was used to produced spherical (slightly RBC-shaped) microparticles of polyether sulfone (PES) with sizes around 10 μm [39], the microparticles were collected in a water bath. The polymer solution was composed of polyether sulfone (PES) and dimethyl sulfoxide (DMSO), and the hydrophilic polyethylene glycol (PEG) was added to regulate and control the microspheres structure, obtaining a slight RBC-shape with a sponge pores internal nanostructure. When the solution concentration was 7.6 wt% in DMSO, and PES/PEG proportion was 10:5 (*w*/*w*), the resulting microspheres were the most similar to RBCs.

Figure 1d Layer-by-Layer [40,41,42,53]: (I) adsorption of negatively charged first polymer chains onto a positive charged template, (II) adsorption of the positive charged second polymer, (III) extraction of the excess of polyelectrolyte using centrifugation and washing, (IV) particle with the desired number of layers repeating the I–III process, (V) decomposition of the core using a low pH solution, and (VI) final polyelectrolyte hollow shell. In Ref. [41], the authors used 6.7 μm Ca(OH)_2_ particles with biconcave discoidal morphology (obtained by chemical reaction) as templates to produce RBC-like microcapsules. Polyallylamine (PAH, dissolved in methanol) and glutaraldehyde (GA, dissolved in ethanol) were chosen as the building block and crosslinker, respectively. After the assembly of the layers of PAH, the ${\mathrm{Ca}(\mathrm{OH})}_{2}$ templates were dissolved in a HCl solution of pH 3 to yield the hollow capsules. On the other hand, in Ref. [42], they made use of mesoporous silica (MS) template particles (Separon SGX 200, average diameter 7.5 μm, pore size 20 nm, available on the market), and 8-arm-PEG functionalized with amine (8-arm-PEG-${\mathrm{NH}}_{2}$) and succinimidyl carboxyl methyl ester (8-arm-PEG-NHS) as building blocks, to fabricate hydrogel PEG microparticles with diameters around 7–9 μm. Figure 1e atom transfer radical polymerization-mediated continuous assembly of polymers $CAP_{ATRP}$ [43] (a specific LbL technique): (I) negative charged templates are incubated in ATRP macroinitiator solution, (II) production of hyaluronic acid (HA, a natural biocompatible polysaccharide) layer by the dispersion of the initiator-functionalized particles in an aqueous stock solution, mainly containing HA and 2-aminoethyl methacrylate hydrochloride (AEMA), (III) template removal by the mixing of the particles in ammonium fluoride ${\mathrm{NH}}_{4}$F buffered HF. The authors used this method with two different initial templates, ${\mathrm{SiO}}_{2}$ solid particles (7 μm in diameter) and mesoporous silica (MS) particles (around 7.2 μm in diameter), producing HA capsules of 7 μm (hollow interior) and HA particles of 6–8 μm (structured interior), respectively, sizes similar to RBCs.

In several references [50,54,55], commercialized spherical quasi-rigid microparticles of polymethyl methacrylate (PMMA) of around 6 and 10 μm in diameter (Spheromers^®^ CA, Microbeads AS) were used to try to mimic RBCs. However, as it can be expected, the rigidity of these particles makes them unsuitable for this purpose. Figure 1f liquid–liquid flow-focusing with a hypodermic needle [44,45,46,56,57]: (I) polydimethylsiloxane (PDMS) precursor, formed by a base of vinyl-terminated siloxane oligomers (Part A) and a curing agent of siloxane oligomers and catalyst (Part B), is injected through a hypodermic needle and a liquid microjet is steadily ejected thanks to the action of a coflowing viscous liquid stream, and (II) microjet breakup into droplets (which are later cured by heat). Since 2016 [44], the interest in the production of monodisperse particles of PDMS has grown significantly due to the remarkable properties of this polymer, among others: flexibility, biocompatibility, and permeability to gases. In Refs. [44,45,46], the authors used the following mixing ratios (Part A:Part B): 6:4, 8:2, and 10:1 to produce spherical particles of around 7 μm in diameter. Figure 1g electrospray with solvent diffusion [47]: (I) two types of solvents, 20 vol% of evaporable solvent (ethanol), and 30 vol% of diffusible solvent (dimethyl sulfoxide, DMSO) were added to the chitosan aqueous solution that was used as the liquid for electrospraying (spray liquid) to form charged chitosan droplets, (II) evaporation of the evaporable solvent (red stars) due to the high temperature of the droplet, (III) phase separation between the diffusible solvent (green circles) and chitosan polymers (blue curved lines), (IV) diffusion of diffusible solvent to the surface in contact with the oil collection solution (yellow fluid) due to the intersolubility, (V) diffusion of the diffusible solvent into the oil collection solution while the cross-linker (terephthalaldehyde, pink curved lines) diffuses into the droplet reacting with chitosan polymers, and (VI) particles with concave morphologies (around 7.4 μm in size) like RBCs obtained by the diffusion of the diffusible solvent and the complete reaction of chitosan with the cross-linker. As reported previously by the same authors, when the spray liquid did not contain evaporable solvent and diffusible solvent, spherical chitosan microspheres with sizes similar to RBCs were obtained [58].

Figure 1h lipid film hydration [48,49]: (I) addition of powered lipids (soybean lecithin) to an organic solvent (chloroform), (II) lipid cake resulting from freezing of the initial solution, (III) addition of the aqueous solution including water-soluble drugs, (IV) production of large multilamellar vesicles by hydration and agitation of the mixture, and (V) final small unilamellar vesicles after sonication and extrusion through polycarbonate membranes (pore size of 8 μm) to improve the monodispersity of the vesicles’ diameters. The giant unilamellar vesicles (GUVs) presented sizes around 6 μm. Figure 1i two-syringe membrane emulsification [50]: back-and-forth manual movements produced an emulsion of PDMS/ water (with SDS surfactant) phases, the droplets were thermally cured into spherical solid microparticles of around 9 μm, reaching a high production rate (1 g/h). The authors used several mixing ratios of PDMS (10:1, 15:1, and 30:1) to check the influence on the mechanical behaviour, also comparing the results with PMMA particles mentioned previously, and with commercialized spherical quasi-rigid microparticles of polystyrene (PS) of around 11 μm in diameter (Dynoseeds^®^ TS 10, Microbeads AS AS). In the same way, as in the case of PMMA particles, the rigidity of PS particles makes them unsuitable to mimic the behaviour of RBCs. Figure 1j 512-channel geometric droplet-splitting microfluidic device combined with a post array part [51]: (I) premixing solutions, dispersed phase (PLGA dissolved in dimethyl carbonate, DMD, at 2 wt%) and continuous phase (polyvinyl alcohol, PVA, dissolved in deionized water at 2 wt%), (II) injection of premixed emulsion through the device, and (III) collection under stirring to remove solvent from the microspheres, satellites were filtered during the collection process by using a sieve. The droplets were polymerized/solidified by the evaporation of the solvent, resulting in spherical PLGA particles of around 9 μm in diameter. Figure 1k premix membrane emulsification [52]: (I) vortex premix emulsification of the continuous phase (water with sodium dodecyl sulfate (SDS) surfactant) and the dispersed phase (PDMS precursor, mixing ratio 6:4), (II) membrane emulsification of the previous mixture passing it through a membrane with a pore size of 10 μm (three times), and (III) thermal curing, obtaining spherical PDMS particles with diameters around 7 μm. This membrane emulsification method allowed for reaching a high production rate (1 g/h) of PDMS particles (6:4), compared to the liquid–liquid flow-focusing method (0.01 g/h) [44]. To finish, a membrane emulsification method similar to that shown in Figure 1k was used to produce micelles of Brij L4 surfactant in water. Briefly, after a manual premixing of the surfactant (typically 1 wt%) and pure water, the emulsion was forced to flow through a precolumn filter having a membrane with an average pore size of 20 μm, resulting in micelles with diameters around 7 μm [59], but with a high polydispersity that needs to be improved.

Most of the methods shown in Figure 1 are complex or have a low production rate of microparticles (see Table 1). This restriction hinders the production of blood analogue fluids with around 45 vol% of microparticles, the typical hematocrit of blood. This drawback will be extended and discussed in Section 3.

**Figure 2:**
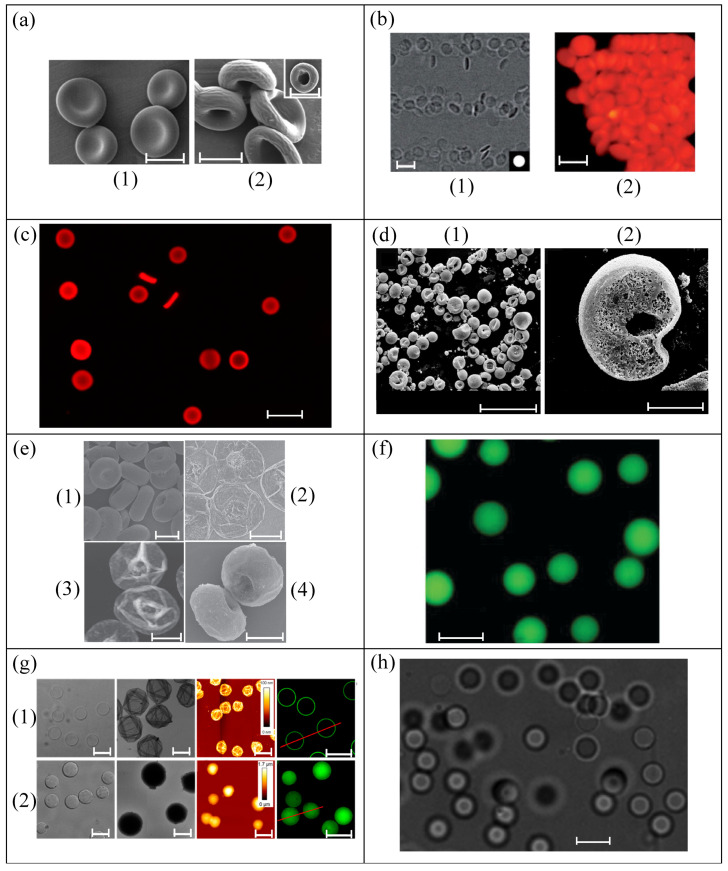

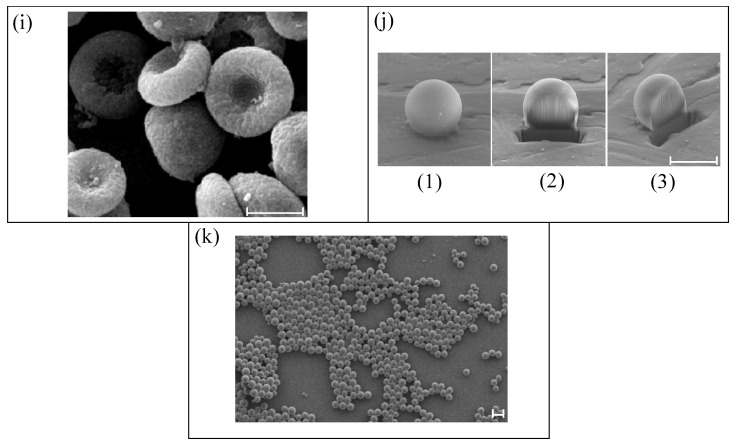
Images of microparticles (RBC templates) produced by using methods from Figure 1: (**a**) electrohydrodynamic jetting [34], (**b**) stop flow lithography (SFL) [35], (**c**) PRINT^®^ [37], (**d**) electrohydrodynamic (electrospraying) [39], (**e**) layer-by-Layer from solid template (LbL) [41], (**f**) LbL from porous template [42], (**g**) $CAP_{ATRP}$ [43], (**h**) liquid–liquid flow-focusing with a hypodermic needle [44], (**i**) electrospray with solvent diffusion [47], (**j**) two-syringe membrane emulsification [50], and (**k**) 512-channel geometric droplet-splitting microfluidic device combined with a post array part [51]. Scale bars are 5 μm in (**a**,**d**-(2),**e**,**g**,**h**), 10 μm in (**b**,**c**,**f**,**h**,**j**,**k**), and 50 μm in (**d**–**1**). Numerals are used to explain, in the text, the information contained by each type of image. Reproduced from Refs. [34,35,37,39,41,42,43,44,47,48,50,51] with permission from National Academy of Sciences, Wiley Periodicals, Inc., WILEY-VCH Verlag GmbH & Co. KGaA, American Chemical Society, AIP Publishing, Elsevier Science INC, and MDPI.

Figure 2 shows images of the microparticles (RBC templates) produced by using methods from Figure 1. Figure 2a (1) RBC-shaped PLGA templates produced by electrohydrodynamic jetting, and (2) final biocompatible microparticles prepared from PLGA templates by LbL deposition of cationic and anionic polymers on the particle surface and subsequent dissolution of the polymer core [34]. Figure 2b shows PEG hydrogel particles obtained by stop flow lithography (SFL) [35], (1) brightfield images from a video of the particles flowing, and (2) fluorescence images of concentrated particles. Figure 2c shows fluorescent images of hydrated HEA microparticles with 5% cross-linker produced using PRINT^®^ [37]. Figure 2d presents scanning electron microscopy (SEM) images of PES particles fabricated via electrospraying: (1) general SEM image of particles from a solution of PES and DMSO 7.6 wt% (PES/PEG, 10:5, *w*/*w*), and (2) SEM image of the cross-section of the porous microsphere obtained from a solution of PES and DMSO 10.6 wt% (PES/PEG, 10:5, *w*/*w*). Figure 2e displays microparticles produced using Layer-by-Layer (LbL) from solid template: (1) SEM (scanning electron microscopy) image of RBC-like Ca(OH)_2_ particles, (2) SEM, and (3) AFM (atomic force microscopy) images of ${\left(PAH/GA\right)}_{10}$ microcapsules after being dried in air, and (4) SEM image of ${\left(PAH/GA\right)}_{10}$ microcapsules dried using the critical point drying (CPD) method [41]. Figure 2f shows a bright-field microscopy image of PEG particles created by LbL coating of MS spheres [42]. Figure 2g, from left to right, differential interference contrast (DIC), transmission electron microscopy (TEM), AFM, and fluorescence microscopy for (1) HA hollow capsules using SiO_2_ templates, and (2) HA replica particles using MS templates, both produced by $CAP_{ATRP}$ [43]. Figure 2h PDMS particles (mixing ratio 6:4) re-dispersed in Dextran 40 produced using liquid–liquid flow-focusing with a hypodermic needle [44]. Figure 2i SEM images of chitosan concave particles prepared with a collection solution containing terephthalaldehyde of 0.1 wt%, produced by electrospray with solvent diffusion [47]. Figure 2j shows SEM images of PDMS (10:1) microparticles produced using the two-syringe membrane emulsification, (1) before and (2) and (3) after being cut by an ion beam for obtaining the cross-section [50]. Finally, Figure 2k presents SEM image of PLGA microparticles produced using 512-channel geometric droplet-splitting microfluidic device combined with a post array part [51].

### 2.2. Deformability

The incredible deformation capacity of RBCs not only allows them to successfully undergo strict contractions, such as a micro-scale stenosis, but also plays a crucial role in the onset of lateral migration of RBCs taking place in microvessels, and therefore in the appearance of the cell free layer (CFL). The lateral migration consists of a shear-induced cell movement toward the channel center, accompanied by tank treading (rotation of the vesicle membrane around its center of mass to stabilize its orientation), shape deformation, and constant inclination [1]. Different measurement techniques have been used to check and study the RBC deformability [60]. Recently, rigid polymethylmethacrylate (PMMA) microparticles were suspended in Newtonian and non-Newtonian solutions to mimic both rheological properties of blood and the cell free layer (CFL) that frequently happens in microcirculation [54]. However, by using rigid microparticles, the CFL was not reproduced faithfully. Hence, deformability or flexibility is a key property of a suitable RBC template. Only a few methods from Figure 1 give rise to this important mechanical capacity. Although really the elastic modulus (*E*) of healthy RBCs is not uniform over the whole cell surface of a RBC (it is higher at the cell center than at the cell boundary [61]), it has been measured/estimated in several references [61,62,63,64,65,66,67,68], by using atomic force microscopy (AFM) [61,63,64,65,66,67,68], and a standard biological Nanoindenter [62]. Under similar experimental conditions, the common average values for *E* were found to be 1.82 ± 0.20 kPa [61], 1.81 ± 0.44 kPa [65], 1.1 ± 0.44 kPa [66], and 1.10 ± 0.40 kPa [63]. However, these values may significantly differ when the storage time of RBCs increased [62,63], varying, for example, from 9.12 to 16.62 kPa in Ref. [63] for 24 h and 48 h, receptively. *E* can also change depending on the protocol used for sample preparation, varying from 1.27 to 7.22 kPa, as reported in Ref. [67], and even it depends on the healthy volunteer, as reported in the supplementary material of Ref. [61]. For all these reasons, in the literature, values of *E* ranging from 1 to 30 kPa were found for healthy RBCs.

**Figure 3:**
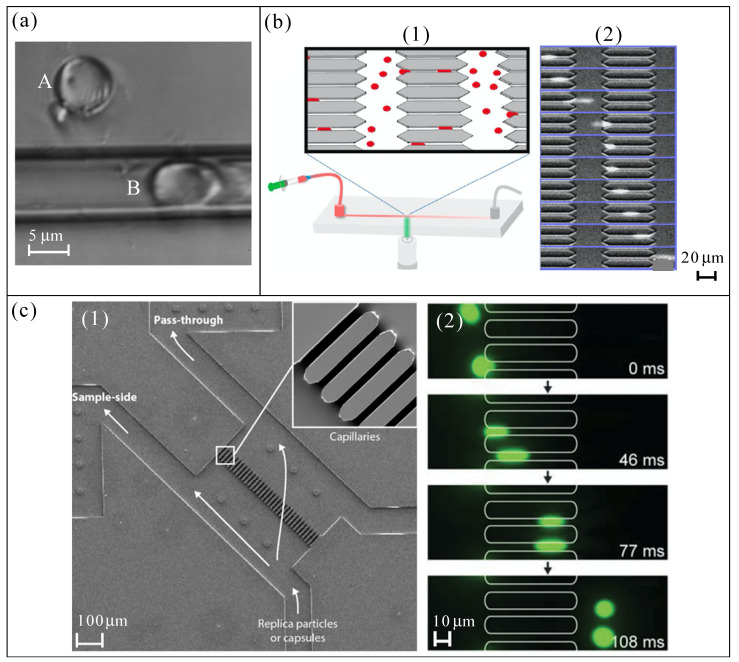

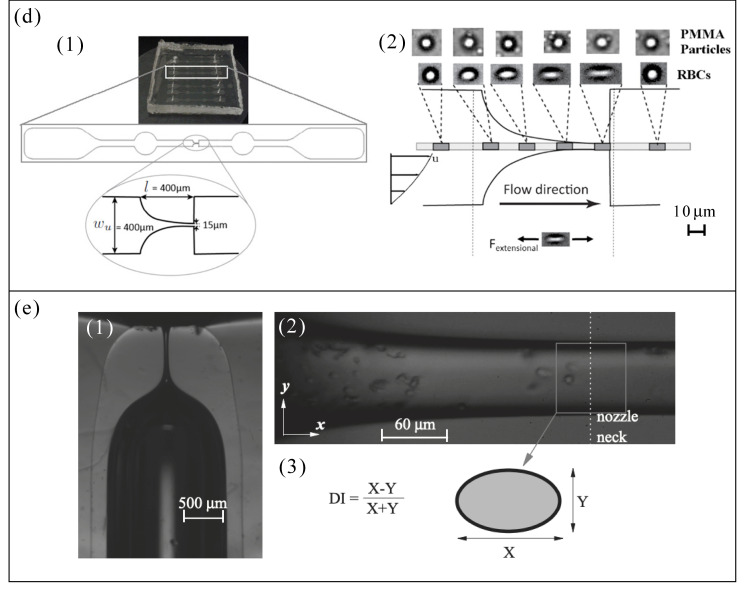
Deformation assessment of microparticles (RBC templates) from the literature: (**a**) PLGA RBC template flowing through a glass capillary (5 μm inner diameter) (A), and outside the capillary (B) [34]; (**b**) illustration of the silicone microfluidic device packed with constricted pores (1), and image sequence showing how a single HEA hydrogel particle passed through a constricted pore (31 ms of time lapse between the frames) (2) [38]; (**c**) SEM image of the PDMS microfluidic device with the possible flow paths (1), and time-lapse fluorescence microscopy images of PEG hydrogel particles passing through the constrictions [42,43]; (**d**) picture and illustration of the PDMS microfluidic device with a hyperbolic microchannel (1), and an example of deformation of PMMA particles and RBCs [54]; (**e**) the whole geometry of the glass axisymmetric micronozzle used (1), visualization of microparticles flowing through the glass micronozzle (the dotted line points out the nozzle neck) (2), and deformation index (DI) definition (3) [50]. Reproduced from Refs. [34,38,42,43,50,54] with permission from National Academy of Sciences, American Chemical Society, WILEY-VCH Verlag GmbH & Co. KGaA, MDPI, and Elsevier Science INC.

The PLGA RBC templates from Ref. [34] were found to be flexible owing to the dissolution of the PLGA core. The elastic modulus *E* of these RBC templates was measured using atomic force microscopy (AFM), obtaining a value of 92.8 ± 42 kPa, four orders of magnitude lower than that of PLGA particles (1.6 ± 0.6 GPa) and near order of magnitude to that of natural RBCs. The flexibility of these templates was confirmed visualizing the stretching when they were injected through narrow glass capillaries (5 μm inner diameter, see Figure 3a). Anywise, further detailed studies about deformability should be done to gain further insight into the mechanical behaviour of these RBC templates. Hydrogel PEG RBC templates from Ref. [35] were tested by flowing through a microfluidic channel containing several constrictions (4 μm in size, similar to that of Figure 3b) under a known pressure difference, they bent rather than stretched. The percentage of particles passing through the constrictions was analyzed, obtaining good results, but the deformability of these RBC templates was not studied deeply. Hydrogel HEA and TEGA RBC templates from Refs. [37,38] demonstrated to have the ability to deform when they flow through a microfluidic device with an array of constricted pores (3 μm in size, see Figure 3b). Although the value of *E*, obtained by using a universal testing machine (tensile testing), for both types of particles was around 7 kPa, further studies are needed to elucidate more details about this ability. In the case of PES RBC template from Ref. [39], their capability to deform was not assessed, but it is expected to be negligible because PES material possesses a *E* ≈ 2.6 GPa.

The deformation and recovery ability of PAH+GA RBC templates was demonstrated by squeezing these capsules through a glass capillary with an inner diameter tapered from 0.84 mm to 5 μm [41]. Its discoidal shape, hollow structure, and good elasticity of the capsule wall (elasticity modulus *E* on the order of hundreds of MPa) allow the deformation and the recovery after the liberation from the microcapillary constriction. However, no comparison was done with human RBCs. The elastic modulus *E* of the PEG RBC templates from Ref. [42] was measured using liquid colloidal probe atomic force microscopy (CP-AFM), obtaining values ranging from 0.2 to 3.3 kPa, depending on the amount of cross-linker used. It was demonstrated that *E* increased with increasing cross-linking concentration. Therefore, it was possible to reach *E* values similar to the typical values for human RBCs. Moreover, these particles showed reversible elastic deformation in a microfluidic blood capillary model (see Figure 3c). Experiments with PEG particles with different cross-linking densities, and with RBCs, were done in the microchannels to calculate the ratio of the number of particles that passed through the microchannels to the number of particles that did not, quantifying its capacity to deform. PEG particles with an *E* of 0.2 kPa showed similar results to RBCs. One year later [43], the same research group/authors investigated the deformability of HA RBC templates depending on the internal structure (hollow or no-hollow) by using the same aforementioned techniques, CP-AFM and the microfluidic blood capillary model [42] (see Figure 3c). These HA capsules/particles manifested tunable stiffness that increases with the increase of the number of layers/steps used in their manufacture. Capsules stiffness ranging from 4.6 to 13.6 mN $m^{-1}$ (*E* from 8.3 to 24.5 kPa, approximately), and particles stiffness from 2.4 to 21.3 mN $m^{-1}$ (*E* from 4.3 to 38.3 kPa, approximately). Despite the similar values of *E*, the ability of the HA capsules to pass through the capillaries was similar to those of RBCs, while the HA particles (no-hollow) showed serious difficulties to do it. These results demonstrate the importance of the internal structure of the microentity on the deformability in a microfluidic device.

Despite the commercialized quasi-rigid particles of PMMA used in Refs. [50,54,55] have an *E* of around 3 GPa, their deformability was also studied in microchannels. Specifically, the 2D PDMS device with a hyperbolic microchannel [54,55], and 3D glass axisymmetric micronozzle [50,69], related to the images display in Figure 3d,e, respectively. Both converging microchannels are based on the same principle: to establish a controlled quasi-homogeneous extensional flow through the contraction (constant strain rate) to produce the elongation (deformation) of the microparticles. The parameter used to measure de deformation capacity was the deformation index (DI), as it is defined in Figure 3e. In all the cases, PMMA particles presented insignificant DI (very small), comparing the results with those of RBCs or flexible particles of PDMS. The DI of the PDMS particles used in Refs. [44,45,46], with an *E* around 1300 kPa, was assessed by using the previously mentioned method depicted in Figure 3d. These PDMS particles (mixing ratios of 6:4, 8:2, and 10:1) resulted to have a value of DI closer to pathological RBCs than healthy ones. Thus, they are not totally valid to replicate healthy RBCs, but this ability could improve modifying suitably the mixing ratio, as it will show later. The deformability of chitosan RBC templates presented in Ref. [47] was not studied, but the *E* of around 9 MPa of this material predicts a low capacity to deform for this type of particles.

The deformation capability of the GUVs (RBC template) [49] was assessed in a hyperbolic-shaped microchannel under a homogeneous extensional flow (similar to that shown in Figure 3d). The DI of the GUVs was a little bit higher than that for RBCs; nevertheless, the agreement was reasonably good. The deformability of PDMS particles (10:1, 15:1, and 30:1), and PS particles, from the Ref. [50] were studied by using the 3D glass axisymmetric micronozzle mentioned previously. While PS particles presented a negligible DI (*E* of around 3 GPa), the deformation capability of the PDMS particles was relevant and depended remarkably on the mixing ratio of PDMS. For the case of PDMS with a mixing ratio of 30:1, *E* was around 90 kPa and DI was 1.6 times higher than the DI value for a mixing ratio of 10:1 (*E* around 1300 kPa). Thus, the capability to deform of PDMS particles is tunable by means of the mixing ratio of PDMS precursor, and other mixing ratios of PDMS might allow one to reach the mechanical behaviour exhibited by real RBCs. Note that PDMS 45:1 or 60:1 showed a *E* of around 18 and 3 kPa, respectively [70] (supplementary material of that reference). In spite of the deformability of the spherical PLGA RBC template proposed in Ref. [51] not being assessed, it is expected to be insignificant because *E* ≈ 4300 MPa. Finally, in the case of the Brij L4 micelles [59], the authors examined the DI of these RBC templates by using a device similar to that showed in Figure 3d. The micelles DI was similar to that of RBCs, a little bit lower than that of RBCs in the contraction region, 0.21 vs. 0.26, respectively. The deformability of the PDMS (6:4) RBC templates from Ref. [52] was also studied by using a hyperbolic-shaped microchannel similar to that shown in Figure 3d. The results of deformation index were similar to those of their counterpart from Refs. [44,46], with an *E* around 1300 kPa.

As it can be seen (Table 1), in most of the cases/references, a direct comparison to the deformability behaviour of RBCs in microfluidic channels was missing. Macroscale, nanoscale, or qualitative measurements of the elasticity (*E*) should be confirmed with in vitro microscale measurements, in a physiologically relevant flow environment, to obtain a more complete understanding of the deformability of the microparticles.

On the other hand, it should be pointed out that, for several polymers, such as PEG [42] or PDMS [50], the deformation capacity and the elastic modulus can be tuned through the cross-linking concentration, being able to decrease the elastic modulus value in a simple way. Lowly cross-linked particles could more easily deform inside the microchannels.

### 2.3. Biological Functionalities

As it was mentioned previously, RBCs contain hemoglobin (Hb), which confers the ability to take/bind, carry, and deliver oxygen from the lungs to all the body tissues, and to do the same with the waste product of metabolism (carbon dioxide) to the lungs, where it is excreted. Under specific treatments, some RBC templates shown in this manuscript could gain this extraordinary biological functionality, among others.

**Figure 4:**
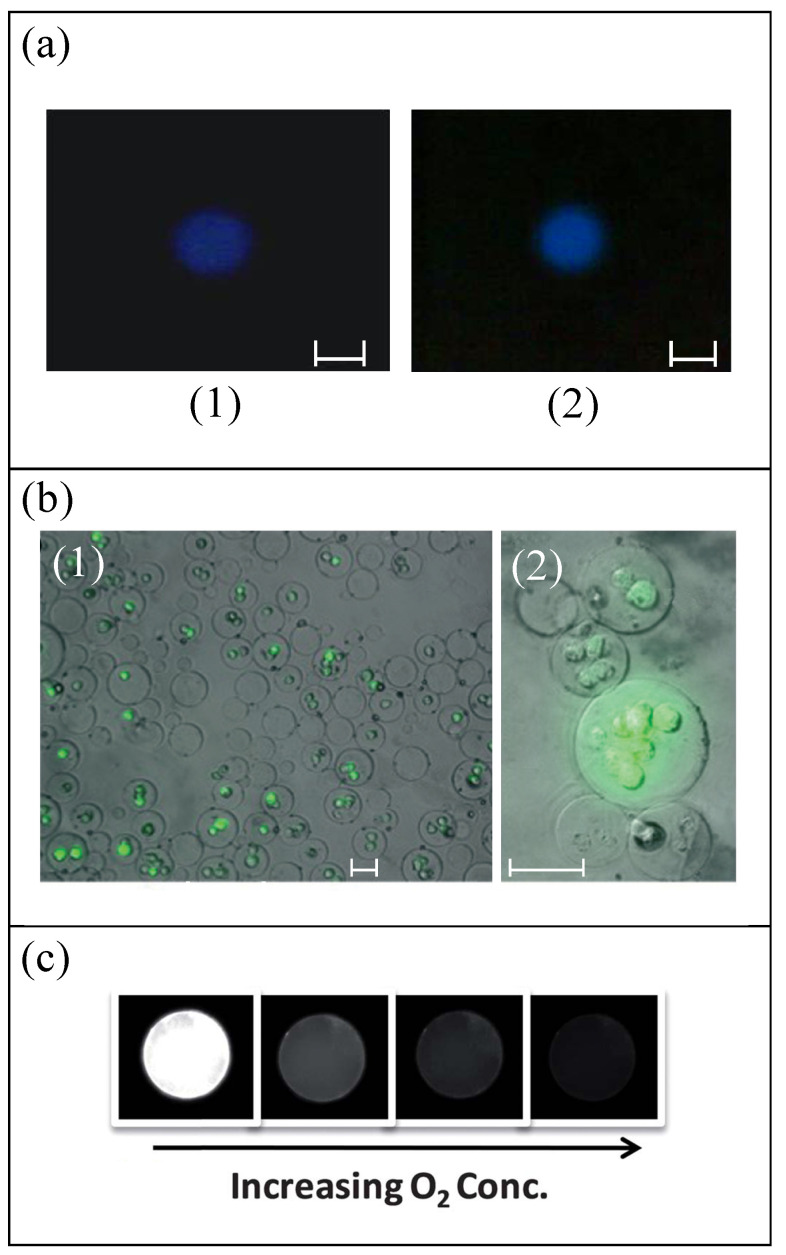
Illustration of biological functionalities of RBC templates. (**a**) chemiluminescence experiments showing the oxygen carrying capacity: (1) PLGA RBC template (2) mouse blood [34], the scale bar is 0.5 cm; (**b**) MDA-MB231 cells encapsulated in PEG microparticles: (1) at day 0 and (2) at day 13 [71], green fluorescence represents live cells, the scale bar is 50 μm; (**c**) phosphorescence intensity of a PtTFPP-bearing PDMS microbead (150 mm diameter) at various oxygen levels [72]. Reproduced from Refs. [34,71,72] with permission from National Academy of Sciences, and The Royal Society of Chemistry.

In Ref. [34], PLGA, a biocompatible and biodegradable copolymer, was used as base material to produce RBC templates. The oxygen carrying capacity of this PLGA RBC template, containing uncrosslinked Hb, was assessed and confirmed, see Figure 4a. These microparticles can also be loaded with drugs by incubation for their controlled release. The drug delivery functionality was tested and demonstrated for dextran and therapeutic drug heparin. The potential application in medical imaging of these microparticles was studied too. For this purpose, iron oxide nanocrystals with an average diameter of 30 nm were encapsulated inside the PLGA particles produced by electrohydrodynamic jetting, making the particles suitable as contrast agents for magnetic resonance imaging (MRI). The requirement of homogenous dispersion of the iron oxide nanocrystals in the PLGA matrix was checked by using transmission electron microscopy (TEM). The PEG particles from Ref. [35] were functionalized with a DNA probe and antibody against epithelial cell adhesion molecule (EpCAM), showing the potential of these particles for in vivo applications, but the applications were not developed. Anyway, other authors [71] developed the production of biocompatible PEG microparticles with cell microencapsulation with high cell viability, as can be seen in Figure 4b. The authors of Ref. [37] checked the circulation and biodistribution of HEA hydrogel particles after intravenous injection, thanks to the fluorescence of the particles, but biological functionalities of these type of RBC template are not developed in this work. One year later [38], using the same production method, TEGA with CEA microparticles loaded with Hb were fabricated to potentiate oxygen transport. The RBC-like PAH+GA capsules fabricated in Ref. [41] were endowed with oxygen-binding and release capacity by assembling additional Hb layers.

In spite of the biological functionalities of PDMS particles not being studied in Refs. [44,45,46,50,52], they were explored by other authors for spherical PDMS particles with sizes much smaller (around 1 μm) [73] and higher (around 80 μm) [72] than those of RBCs. PDMS microbeads have been demonstrated to have potential use as discrete oxygen sensors [72], see Figure 4c, and for the delivery of reporter genes into cultured animal cells [73]. Chitosan RBC templates [47] displayed acid-triggered dissolution and auto-fluorescence, which makes them very promising for biomedical applications, but these applications were not developed in this study.

On the other hand, the interaction between synthetic microparticles and biological systems has also been an important area of research in the last several years; for example, the development of artificial oxygen carriers to substitute RBCs in the body [74,75,76,77,78], but it is beyond the main scope of the present review focused on blood particulate analogue fluids for in vitro studies.

Table 1 summarizes the information presented in Section 2.

## 3. Dynamic Flow of Particulate Fluids

This section discusses and reviews the most appropriate rheological measuring techniques used to validate whether or not: (i) The proposed blood particulate analogue fluid shows a reliable rheological functionality (i.e., rheological shear-thinning behaviour, extensional flow characteristics, aggregation) in comparison with real blood; (ii) A similar flow phenomenon such as cell free layer (CFL) is reproducible in comparison to real blood under the same in vitro flow conditions.

Blood particulate analogue fluid is a viscoelastic fluid mixture that should closely match the whole characteristic behaviour for real blood. In this way, it is important to take into account the rheological behaviour of such analogue fluid’s dependence on the combined contribution of its carrier fluid and its suspended particulate. The carrier fluid should mimic the characteristic behaviour of the blood plasma, while the suspended particulate should match the physical properties of the most abundant blood cells, i.e., the RBCs. It was reported in Refs. [24,79] that blood plasma has a slight elastic behaviour caused by a specific protein called fibrinogen [79]. To understand the important role of blood plasma, its rheological behaviour under steady shear, and extensional flow measurement will also be discussed in this section.

Table 2 summarizes the studies developed by several authors [29,38,44,46,49,52,54,55,59] to characterize the blood particulate analogue fluids. The table includes detailed information about the particulate fluids studied: particles, carrier fluid (liquid as plasma), particle concentration, as well as the measurements developed for each particulate fluid.

### 3.1. Rheology: Shear and Extensional

The rheological characterization consists of the study of the blood particulate analogue fluid under both shear (steady, oscillatory) and extensional flow. The shear flow measurement was performed by using a rotational rheometer equipped with either a plate–plate, a cone–plate, or even a serrated plate–plate geometry, while for extensional flow a capillary-breakup extensional rheometer was used.

#### 3.1.1. Shear Flow Measurement: Steady and Oscillatory

Steady shear flow is reported as a common measuring technique to characterize the behaviour of the solution viscosity, usually in the range of a shear rate of 1 ≤$\dot{\gamma }$ (s${}^{-1}$) ≤ 10${}^{4}$ [29,44,46,49,52,54,55,59]. The carrier fluid may either be a Newtonian-like or non-Newtonian, with either quasi-rigid [29,54,55] or flexible [38,44,46,49,52,59] microparticles. In the literature, several authors have used dextran 40 (Dx40) as a carrier fluid, where Dx40 is a water soluble polymer. As a result, under steady shear flow, there are some interesting common observations, such as: (i) if a solution of Dx40 is used with no particles, the solution behaves as Newtonian-like fluid with constant viscosity [29,44,46,54,55]; (ii) if rigid particles are added to Dx40, viscosity increases, but it remains Newtonian without affecting elasticity [54,55], (iii) if flexible particles are added to Dx40, the solution tends to display a smooth shear-thinning behaviour at low shear rate up to a certain value, which then tends to behave as a Newtonian-like fluid at high shear rates [44,46,59]; (iv) if an additional polymer is added to Dx40 (in case (ii) or (iii)), the solution exhibits a shear-thinning closer to behaviour shown by a solution with RBCs, or blood [54,55].

Rheological measurement was first conducted by Fukada et al. [29] to characterize the behaviour of particulate fluid based on suspended particles of polystyrene (PS) at 12 wt% prepared in three aqueous solutions: of calcium chloride (${\mathrm{CaCl}}_{2}$) at a molecular weight of 10 mM, of 5% dextran 70 (Dx70), and a solution of ${\mathrm{CaCl}}_{2}$ at 10 mM and 5% Dx70. The results have shown that first and second solutions have a constant viscosity under steady shear flow, whereas the addition of ${\mathrm{CaCl}}_{2}$ to Dx70 in the third solution allows the solution to behave as a non-Newtonian fluid with a shear thinning behaviour similar to blood behaviour. Additionally, in case of having a solution of only ${\mathrm{CaCl}}_{2}$ with particles at 32 wt%, the results have shown that the variation of the ${\mathrm{CaCl}}_{2}$ molecular weight (Mw) from 10 to 30 mM allows the viscosity curve to change from a Newtonian-like (Mw = 10 mM) fluid to a non-Newtonian fluid (Mw = 20, and 30 mM) behaviour.

Chen et al. [38] developed the deformable TEGA (hydrogel) microparticles described in Section 2, loading them with bovine hemoglobin (Hb, concentration of 5.2 g/dL). These TEGA particles were suspended in phosphate buffer saline (PBS) to form a blood analogue fluid with a particle volume fraction of 40%. The rheology was examined under steady shear flow and compared against the curve of mouse blood with the same RBC volume fraction. The resulting curves did not match very well, and this blood analogue fluid showed values of viscosity lower than that of the mouse blood. In addition, it should be mentioned that the viscosity curve for mouse and human blood are very different from each other; in the case of mice, the values of viscosity are much lower for high shear rates.

**Figure 5:**
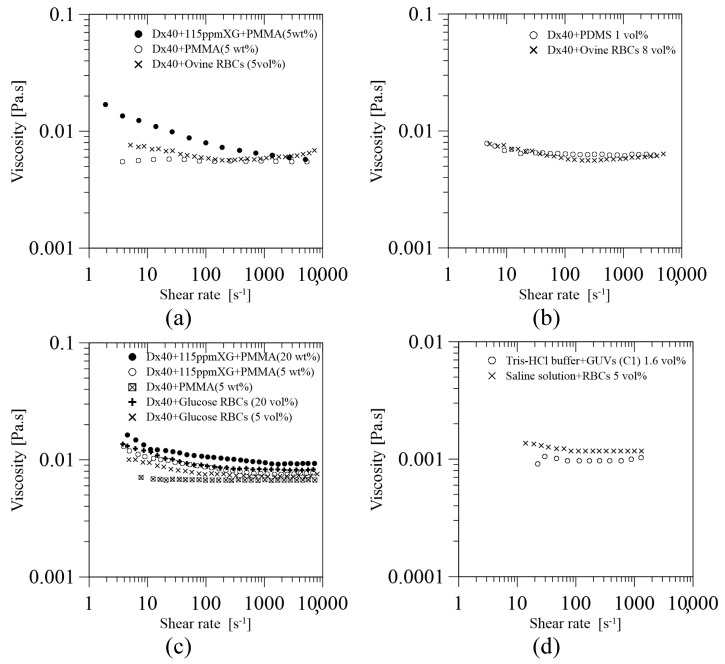
Steady shear viscosity curves as a function of the shear rate obtained by: (**a**) Calejo et al. [54], (**b**) Carvalho et al. [49], (**c**) Pinho et al. [55], and (**d**) Muñoz-Sánchez et al. [44].

Calejo et al. [54] proposed two blood particulate analogue fluids: a Newtonian-based and a non-Newtonian-based solution. The Newtonian one was made of Dx40 with a quasi-rigid particulate of polymethylmethacrylate (PMMA) of 5 wt%, while the non-Newtonian one was prepared by adding a viscoelastic polymer of xanthan gum (XG) of 115 ppm to the previous solution. The rheological behaviour for both particulate fluids under steady shear flow is presented in Figure 5a in comparison with ovine RBCs of 5 vol% suspended in Dx40. The Newtonian-based solution shows as constant viscosity, while the non-Newtonian-based solution exhibits a viscoelastic shear thinning behaviour at a low shear rate, and a constant viscosity at high shear rate. The results have shown that the addition of the polymeric fluid conferred a certain degree of elasticity, which allowed for mimicking the viscosity behaviour of ovine RBCs in Dx40. As can be seen, rigid microparticles in a Newtonian-based solution are not able to provide elasticity to the solution by themselves; it is necessary to use another additive.

Muñoz-Sánchez et al. [44] have introduced for the first time the use of flexible polymer microparticles with a high degree of monodispersity. These particles are made of polydimethylsiloxane (PDMS) at a proportion of a base to curing agent of 6:4. The rheological measurements show that the use of 1 vol% of cured PDMS particles suspended in Dx40 confers a smooth shear thinning behaviour at low shear rate, while a constant viscosity at high shear rate. This result is in good agreement with the rheological behaviour obtained from a solution containing ovine RBCs suspended at dextran 40 at 5 vol%, see Figure 5b.

Pinho et al. [55] extended the work of Calejo et al. [54] by using an analogue fluid based on quasi-rigid microbeads made of PMMA suspended in a viscoelastic carrier fluid made of a solution of Dx40 and XG (115 ppm). Two concentrations of PMMA, 5 and 20 wt%, were studied. The experimental results, shown in Figure 5c, show that the shear-thinning behaviour of the viscosity curves improves by the addition of XG 115 ppm to the solution of Dx40 with 5 wt% of PMMA particles, as it was mentioned previously. Additionally, the viscosity curves for the viscoelastic solution with PMMA microparticles, 5 and 20 wt%, suspended in Dx40 and XG 115 ppm show a rheological behaviour close to that obtained for the glucose-rich RBCs, representing the pathological RBC, for 5 and 20 vol%, respectively.

Carvalho et al. [49] developed a blood particulate analogue fluid containing the lipid particles (GUVs) described in Section 2, suspended in a buffer solution of Tris-HCl (i.e., a solution of Trizma^®^ base and hydrochloric acid). The proposed GUV solution under steady shear flow shows a rheological behaviour similar to the one obtained for RBCs of 5 vol% in saline solution, as it can be seen in Figure 5d. This was the first work in the literature introducing the use of flexible GUVs particles to mimic the flow behaviour of RBCs, which still require further developments and improvements as suggested by the authors [49].

**Figure 6:**
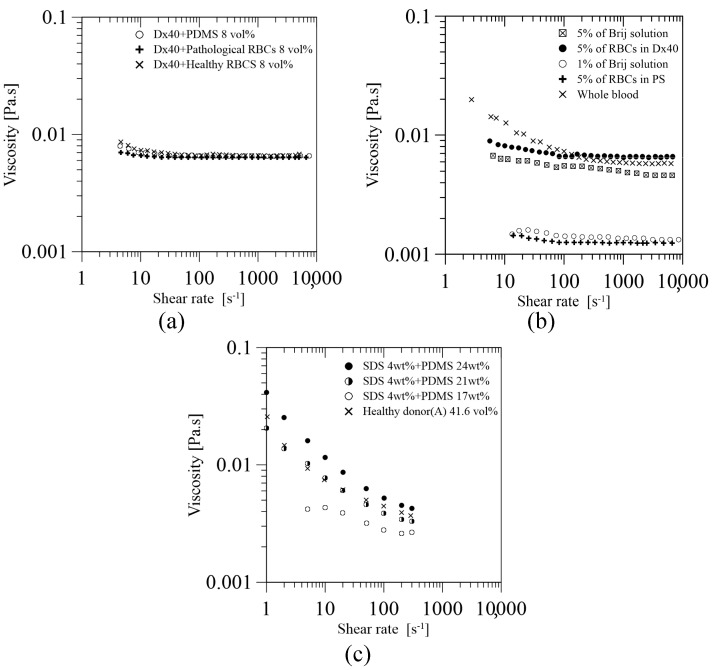
Steady shear viscosity curves as a function of the shear rate obtained by: (**a**) Pinho et al. [46], (**b**) Carneiro et al. [52], and (**c**) Lima et al. [59].

Pinho et al. [46] extend the work done in [44] to study the use of PDMS microparticles with different crosslinking ratios of 10:1, 8:2, and 6:4. As these particles showed a similar deformation index (DI), they prepared an analogue fluid by mixing these particles (8 vol%) in Dx40. This analogue fluid results in a steady shear viscosity curve with a smooth shear thinning behaviour close to those curves obtained for healthy and pathological RBCs at 8 vol% suspended at Dx40, see Figure 6a.

Lima et al. [59] developed a simple multiphase blood analogue fluid by using the Brij L4 surfactant micelles mentioned in Section 2, suspended in pure water. The viscosity curves under steady shear flow of this fluid were compared to different solutions with RBCs and to the curve of human whole blood; see Figure 6b. The results show that the curves of this blood analogue fluid, with concentrations of 1 and 5 wt% of micelles of Brij L4, have a reasonable agreement with the ones obtained from the solutions of RBCs with 5 vol% suspended in physiological saline (PS) and Dx40, respectively. Additionally, at high shear rates, the viscosity curve of the 5% of Brij L4 micelles and the human whole blood is in good agreement, despite the fact that the HCT% is very different. More studies should be done with this fluid to assess and confirm its use as blood analogue fluid.

Carneiro et al. [52] have developed an analogue fluid made of deformable PDMS (6:4) microparticles, mentioned in Section 2, suspended in an aqueous solution of 4 wt% sodium dodecyl sulfate (SDS). The proposed solution at a PDMS concentration of 21 wt% at 20 °C has shown to have a shear-thinning behaviour with a viscosity curve similar to that obtained for the whole blood of 41.6 HCT% (haematocrit) at 37 °C, see Figure 6c. This achievement suggests that this fluid has an enormous potential to be a blood analogue fluid for in vitro studies, but several issues should be improved, among others: the flexibility of the particles is still far away from that of healthy RBCs, the concentration of microentities is quite different, and the properties of the base fluid are different from real plasma.

Oscillatory shear flow measurements were only performed in three works [29,52,55]. Fukada et al. [29] obtained a Newtonian-like solution, while, in Refs. [52,55], it was as a viscoelastic-like solution. The oscillatory shear flow curves allow a more complete understanding of the rheological behaviour of the proposed solution, under small or large amplitude oscillatory shear flow, entitled as SAOS and LAOS, respectively. Earlier in 1989, Fukada et al. [29] investigated the oscillatory shear flow curves (i.e., the dynamic viscosity, and the storage and loss modulus) for microspheres of polystyrene suspended in: (i) distilled water at several concentrations of polystyrene particles (12, 24, and 32 wt%); and (ii) in a solution of distilled water having polystyrene particles of 32 wt% with ${\mathrm{CaCl}}_{2}$ at several molecular concentrations (0, 10, 20, and 30 mM). The results have shown that the viscosity in oscillatory flow curves increased with increasing either the particulates concentration, or the ${\mathrm{CaCl}}_{2}$ concentration.

Up to date, there are only two works [52,55] where the rheological behavior of their proposed viscoelastic analogue fluids (defined in Table 2) was studied under SAOS and/or LAOS tests. In particular, Pinho et al. [55] investigated their proposed fluids under both SAOS and LAOS tests, while, in Carneiro et al. [52], it was only analyzed under LAOS testing. The SAOS [55] measurement was carried out within the viscoelastic linear region to measure the storage ($G^{\prime }$) and loss ($G^{\prime \prime }$) moduli. The LAOS [52,55] was used to measure the large-rate ($\eta_{L}^{\prime }$) and minimum-rate ($\eta_{M}^{\prime }$) dynamic viscosities, the large ($G_{L}^{\prime }$) and minimum ($G_{M}^{\prime }$) strain elastic moduli, and the shear-thickening (*T*) and strain-stiffening (*S*) ratios. Further reading about LAOS measuring technique can be found in Ref. [80].

**Figure 7:**
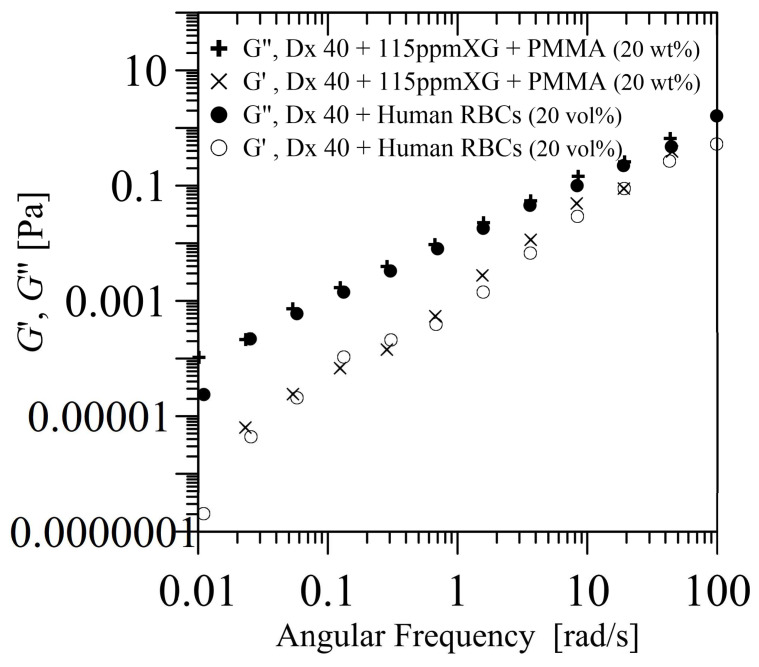
SAOS measurement of the storage ($G^{\prime }$) and loss ($G^{\prime \prime }$) moduli for a solution of PMMA of 20 wt% suspended in Dx40 and 115 ppm XG, and a solution of healthy RBCs of 20 vol% suspended in Dx40 [55].

In Pinho et al. [55], the measurements of the SAOS were performed within a frequency (ω) of 0.01 to 100 rad/s, while the LAOS at an imposed frequency of 0.1 and 1 rad/s, using a direct strain oscillation module. The SAOS measurements revealed a non-negligible elastic contribution for the proposed blood particulate analogue fluid (PMMA suspended in Dx40 and 115 ppm XG), which is dominated by the viscous behaviour, while the LAOS measurements confirmed the shear-thinning behaviour of that fluid. In conclusion, within the linear viscoelastic region, the proposed solution showed a good agreement with the corresponding solution with healthy RBCs. For illustration, Figure 7 shows the SAOS measurement of the storage ($G^{\prime }$) and loss ($G^{\prime \prime }$) moduli of a solution of PMMA of 20 wt% suspended in Dx40 and 115 ppm XG, in comparison with a solution of healthy RBCs, 20 vol% suspended in Dx40. In addition, the analogue solution displays small elasticity in the LAOS results at an angular frequency of 1 rad/s, which was not noticeable at lower frequency (0.1 rad/s). The results obtained for the $G_{L}^{\prime }$ and $G_{M}^{\prime }$, $\eta_{L}^{\prime }$ and $\eta_{M}^{\prime }$, and *T* and *S* indicate a shear-thinning behaviour.

In Carneiro et al. [52], the LAOS was imposed using the direct strain oscillation module at the frequency of 0.158 and 1 rad/s, for strain amplitudes of 1000% and 10,000%. The results suggested that, at 0.158 rad/s, the proposed solution showed a dominant viscous behavior with a weakly elastic nature at both strain amplitudes, while, at 1 rad/s, the solution showed a higher viscoelastic behaviour with a sufficient stored energy similar to the whole blood.

**Figure 8:**
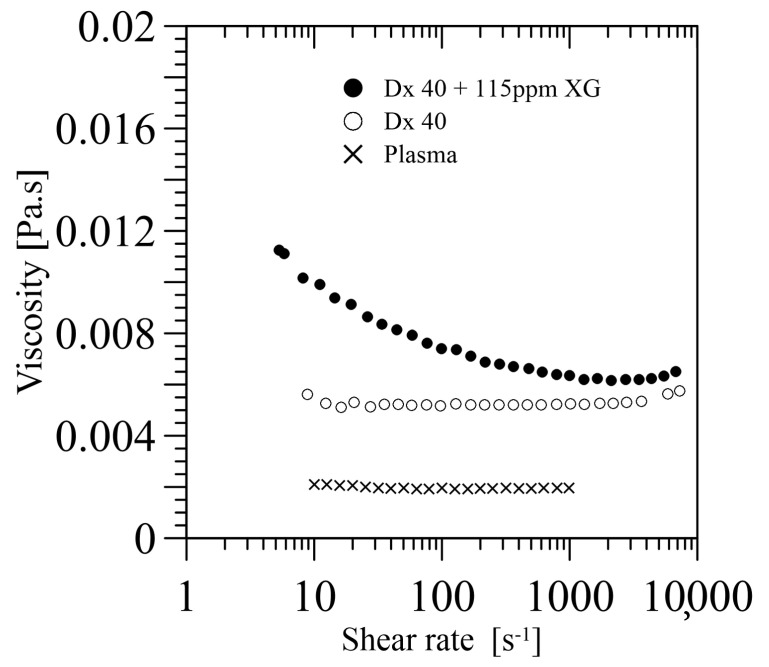
Steady shear viscosity curves as a function of the shear rate obtained by Brust et al. [24] for blood plasma, and Pinho et al. [55] for Dx40 and Dx40+115 ppmXG.

To end this section, it should be pointed out that the viscosity of the carrier fluid used until now, such as water, Dx40, or Dx40+115 ppm XG, etc., does not match that of real plasma at 20 or 37 °C, around 1.9 and 1.3 mPa·s, respectively. Brust et al. [24] showed that human blood plasma under steady shear flow has a Newtonian behaviour with a very low shear viscosity of about 1.95 mPa·s at 20 °C, which completely differs from the carrier fluid viscosity used in the literature, see Figure 8. Future blood particulate analogue fluids should take this issue into account because it could be a key point in multiphase phenomena, among others.

#### 3.1.2. Extensional Flow Measurement

The rheological behaviour under uniaxial extensional flow gives us additional rheological information to those obtained under shear flow conditions. The stress associated with the non-Newtonian fluid stretching, under an extensional flow in a microchannel, can result in a sharp increase of the extensional viscosity. The extensional relaxation time measured by extensional rheology gives us information about the extensional viscosity. Sousa et al. [5] reported that the flow behaviour of blood analogue fluids without particles under the same shear flow conditions may reveal the same rheological behaviour as blood, but that does not entail that the behaviour must be equal under the same extensional flow conditions (or even in microscale flow). For that reason, it is very important to check similar behaviour under pure extensional flow between a proposed blood particulate analogue fluid and real human blood.

The elastic properties of such analogue fluids can be investigated by using a capillary-breakup extensional rheometer (CaBER). This technique is frequently used to measure the extensional relaxation time for viscoelastic aqueous solutions undergoing extensional flow. The fluid sample is placed between two circular plates separated by an initial gap, where under extensional flow the upper plate reached the final height, while the lower plate is kept fixed. The sample undergoes a filament thinning process and the filament diameter D(t) decreases exponentially as a function of time. Thus, the exponential decay rate for the filament diameter is obtained by [81]:

(1) $$\frac{D(t)}{D_{0}}={\left(\frac{GD_{0}}{4\sigma }\right)}^{1/3}\exp\left(-\frac{t}{3\lambda }\right),$$

an equation frequently used for viscoelastic aqueous solutions, where $D_{0}$ is the initial diameter, and σ is the surface tension, while *G* and λ are the extensional elastic modulus and the extensional relaxation time of the viscoelastic fluid, respectively. Then, the relaxation time of the sample is determined from the slope (−t/3λ) of the linear fit to the experimental data of $log[D\left(t\right)/D_{0}]$.

**Figure 9:**
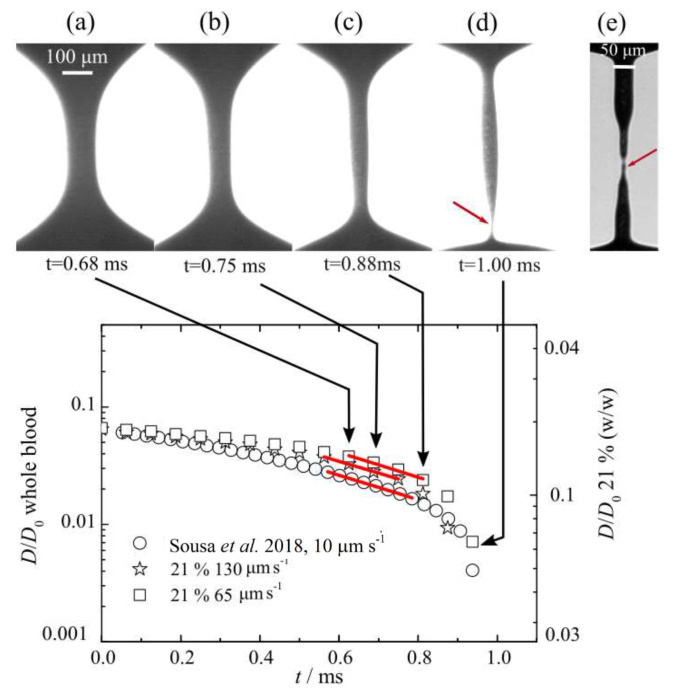
Time evolution of the filament thinning process of the particulate analogue fluid of 21 wt% PDMS microparticles suspension in an aqueous solution of 4 wt% SDS, in comparison with the blood (circles) [52]. The red lines point out the linear fit to obtain the extensional relaxation time λ. Reproduced from Ref. [52] with permission from The Royal Society of Chemistry.

For now, Carneiro et al. [52] have been the only authors reporting in the literature this type of extensional rheology measurements for their proposed blood particulate analogue fluid, which is composed of the PDMS (6:4) particles suspended in an aqueous solution with 4 wt% SDS, as mentioned previously. They have tested several sample concentrations using the slow retraction method (SRM) [82], but only the sample with 21 wt% of PDMS microparticles have shown a realistic agreement in comparison with the data taken from Ref. [81] for real human blood, as presented in Figure 9. After fitting the data in Figure 9, the analogue fluid proposed by Ref. [52] has a relaxation time λ = 310 ± 15 μs, while, for whole blood, λ = 114 ± 30 μs as reported in Ref. [81], both measurements at room temperature (around 21 °C) and surrounded by air. The difference in the relaxation time, or even in the shape of the filament distribution before the filament breakup (Figure 9), is possibly due to:

- The microparticles (RBCs) concentrations which differ from Ref. [52] to Ref. [81], 21 wt% PDMS and 40.3 vol%, respectively.
- The limitation faced by the slow retraction method used, which differs from Ref. [52] to Ref. [81]. The relaxation time in Ref. [52] was determined by a commercialized CaBER device that allows a reliable measurement down to 240 μs, while it was down to 100 μs in Ref. [81]. The extensional device used in Ref. [81] was a custom-made setup developed by Sousa et al. [83] that allows the use of an outer silicone oil bath to avoid evaporation effects, and to visualize the blood cells in the filament. The relaxation time measured using blood samples surrounded by silicone oil was around λ = 259 ± 47 μs. In addition, the fluid sample was stretched at a constant speed down to 10 μm/s, while these data were around 65 μm/s for the commercial CaBER used in Ref. [52].

In conclusion, extensional flow measurement is also a key measuring technique that must be considered when a blood particulate analogue fluids are characterized. In addition, a suitable configuration of this type of device should be used to be able to get reliable measurements for such weakly viscoelastic solutions.

**Figure 10:**
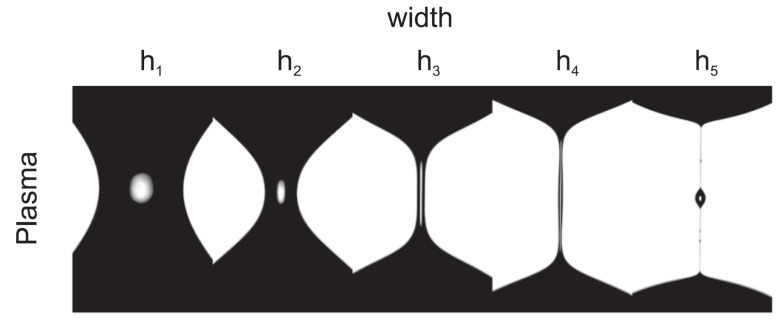
Images of the capillary break extensional experiment [24].

Finally, it should be taken into account in this subsection that human plasma has a slight elastic behaviour caused by a specific protein called fibrinogen [24,79]. It has been demonstrated that, under pure extensional flow, blood plasma has a temperature dependent relaxation time [24]. At around 20°, the values reported for plasma have been 126.7 μm/s [24,79] (surrounded by air), and λ = 42 ± 3 μs (surrounded by air) and λ = 139 ± 15 μs (surrounded by silicone oil) [81]. Figure 10 shows the filament break in a CaBER recorded by using a high speed camera [24]. Varchanis et al. [79] extend the work done in Ref. [24] to investigate numerically the response of human blood plasma under strong extensional flows in a CaBER, and under constriction complex flows in a contraction–expansion microchannel. In both cases, the numerical results were in good agreement with experimental ones. Therefore, an important issue that should not be ignored in future studies is to have a carrier fluid with viscoelastic properties closer to those of blood plasma.

### 3.2. Cell Free Layer

Cell free layer (CFL) or plasma layer is a phenomenon that is related to RBCs flow behaviour, and frequently happens in microvessels for diameters smaller than 300 μm [22]. For blood particulate, analogue fluids may preferably be called “microparticle-free layer”. This phenomenon tends to force the RBCs to undergo an axial migration towards the center forming a thin-depleted layer free of RBCs. In microcirculation and blood-on-chip devices, the CFL is influenced by several multiphysical and hemorheological factors such as cell interactions and deformability, hematocrit, flow rate, viscosity, and geometry of the microvessel or microchannel. This layer is frequently known to occur around the microvessels/microchannels walls, and, as a result, different kinds of microfluidic devices were developed to separate the blood cells from plasma [84]. However, some recent studies have also observed the formation of CFLs in the middle of the channel in networks having converging bifurcations and microbubbles [85,86,87,88,89]. For blood particulate analogue fluids, this phenomenon was only reported by a limited number of authors, Refs. [46,52,54,55], by using quasi-rigid microparticles [54,55], and flexible microparticles [46,52]. Table 2 shows a brief summary with regard to each study.

**Figure 11:**
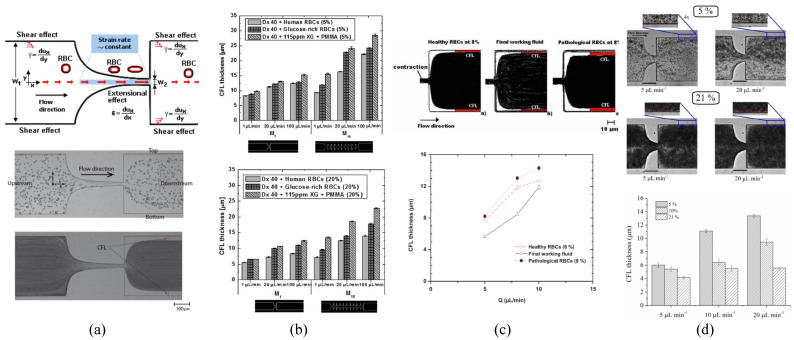
(**a**) Sketch of the flow behaviour in a microchannel with hyperbolic contraction (upper-part), with a particulate fluid of RBCs undergoing a constant strain-rate only along the contraction center-line, with a shear effect only at the microchannel walls [46]. Flow visualization (lower-part) for the CFL thickness analysis using ImageJ software for post-processing with the small vertical red line representing the CFL [54]. CFL thickness analysis downstream of a contraction microchannel for (**b**) three types of particles (PMMA, healthy RBCs, and glucose-rich RBCs) at concentration of 5 wt% and 20 wt%, and with flow rates of 1, 20, and 100 L/min in two different microchannels [55]; (**c**) three types of particles (PDMS, healthy RBCs, and pathological RBCs) with flow rates of 5, 8, and 10 L/min [46], (**d**) three types of particles (PDMS of 5, 10, and 21 wt% concentrations) at the flow rate of 5, 10, and 20 L/min [52]. Reproduced from Refs. [46,52,54,55] with permission from MDPI, AIP Publishing, Elsevier Science INC, and The Royal Society of Chemistry.

In vitro blood experiments have demonstrated that the thickness of the CFL is strongly dependent on the dimensions and geometrical configurations of the microchannel [4,84,85,86]. Accordingly, it was reported that the use of a straight microchannel with a region of a hyperbolic-shaped contraction followed by an immediate abrupt expansion allowed a further enhancement on the CFL. The contraction of the microchannel is designed to impose a constant strain rate, $\dot{\epsilon }$ = d$u_{x}$/d*x*, along the centerline of the contraction, where $u_{x}$ refers to the particle/RBC axial velocity (see Figure 11a upper-part). To differentiate between microchannels with the same geometrical configuration (i.e., a contraction), a dimensionless number known as Hencky strain ($\epsilon_{H}$) was used, defined as the ratio of the microchannel width (straight part) to the width of the throat. Figure 11a upper-part shows that the extensional flow along the centerline of the hyperbolic-shaped contraction is extremely high, while, at the microchannel walls, the shear effect, $\dot{\gamma }$ = d$u_{x}$/d*y*, is less pronounced compared to the extensional effect [90,91,92]. The majority of the cells flow studies have been performed by means of hyperbolic channels to assess the deformability of both healthy [91,93,94] and pathological cells [95,96,97]. For instance, in a recent study by means of hyperbolic converging microchannels, Faustino et al. [97] have measured the deformations and motions of RBCs of end-stage kidney disease patients. However, similar flow extensional studies were also performed by other researchers by using cross slot microchannels [98,99,100]. A comparison between different kinds of strategies to assess the cell deformability can be found at Bento et al. [90]. Regarding the studies performed with blood particulate analogue fluids, all of them have used PDMS hyperbolic microchannels fabricated by soft lithography [46,52,54,55].

The thickness analysis of the CFL for blood particulate analogue fluids is frequently done downstream of the contraction because upstream its measurement is more difficult. The CFL thickness can be determined by using a flow visualization technique, by means of a high speed camera combined with a microscope. The recorded data are post processing in ImageJ [101] to determine the microparticle-free layers and their thickness [86], by using the ImageJ-plugin Z project [52,54,55] or MTrackJ [102,103], see Figure 11a lower-part.

Calejo et al. [54] have investigated the formation of CFL in a microchannel with a hyperbolic contraction of $\epsilon_{H}$ = 3.3 using two different carrier fluids: a Newtonian solution based on Dx40; and a non-Newtonian solution composed of Dx40 and a viscoelastic solution made of XG (115ppm). Both solutions were prepared at the concentrations of 5 wt% of quasi-rigid spherical particles of PMMA. The results have shown that each carrier fluid introduces different tendencies in the CFL thickness, both cases slightly close to that formed by healthy RBCs. The Newtonian base solution tends to form a CFL slightly smaller than that formed by RBCs (suspended in Dx40), in contrast to the non-Newtonian based solution, which is slightly bigger. In conclusion, fluid elasticity for this non-Newtonian solution contributes to the particle migration towards the center of the microchannel, besides an additional contribution that may result from the normal stress difference. In this study, the contribution due to the shear thinning effect on the CFL was negligible due to the use of a Reynolds number greater than 1. Additionally, the thickness of the CFL has shown to increase slightly with the imposed flow rate, which corroborates the results obtained by Rodrigues et al. [85]. Moreover, they corroborated that the PMMA particles had different tendencies to migrate towards the microchannel center depending on the rheological properties of the carrier fluid. In another study performed by Pinho et al. [55], they have extended the work done by Calejo et al. [54] to study the variation of the CFL thickness by using different kinds of microchannels with one-single hyperbolic-shaped contraction ($\epsilon_{H}$ = 3.3), and microchannels with a sequence of ten-hyperbolic-shaped contractions having the same Hencky strain ($\epsilon_{H}$ = 2). In this study, they have used the same carrier fluids and microparticles as applied in Ref. [54], but with particle concentrations of 5 and 20 wt%. The obtained CFL measurements shown in Figure 11b, where the CFL thickness for three different types of particulate fluids is compared: PMMA particles (suspended in a viscoelastic solution), healthy RBCs (suspended in dextran 40), and glucose-rich RBCs (suspended in dextran 40). The results have shown that CFL formation is highly influenced by the changes in the following three parameters: the geometrical configurations of the microchannel, the imposed flow rate, and the particle concentration. Accordingly, the CFL thickness is increased when the imposed flow rate is increased, and when the number of sequential hyperbolic concentrations is increased, while in contrast it tends to decrease when the particle concentration is increased. The results obtained with PMMA particulate fluid have shown a good agreement with both of the healthy and glucose-rich RBCs fluids [55]. In conclusion, a thicker CFL is formed in the sequential hyperbolic concentrations, due to the higher migration of the particles towards the centerline, which is promoted due to the combined effect of the shear-thinning behaviour and elasticity of the viscoelastic solution, besides the contribution of the extensional flow which results due to the sequential number of hyperbolic contraction. However, by using rigid microparticles, the CFL was only formed clearly downstream of a hyperbolic contraction, whereas no CFL was observed at the upstream region. Both studies [54,55] have suggested that the only way to closely mimic some microscale blood-flow phenomena is by using deformable microparticles.

Recently, Pinho et al. [46] have used several types of flexible PDMS microparticles suspended in Dx40. The microparticles were prepared at different mixture ratios of PDMS precursor and curing agent, as shown in Table 2, and generated by using the flow focusing technique described in Section 2; more details are in [44,45]. The results have shown that a thin microparticle-free layer was developed downstream of hyperbolic contraction with $\epsilon_{H}$ = 3.5. A thin layer of the CFL was formed attached to the microchannel walls, and the thickness of this layer tends to increase with the flow rate, as shown in Figure 11c. These results are in good agreement with that of human RBCs (healthy and pathological) suspended in the same carrier fluid, Dx40. More recently, Carneiro et al. [52] have investigated the formation of the CFL in a microchannel with a hyperbolic contraction of $\epsilon_{H}$ = 3.16, using flexible PDMS microparticles produced by a premix membrane emulsification technique. The particle concentrations were 5, 10, and 21 wt%, and Reynolds numbers ranging from 0.08 ≤Re≤ 2.13. The results have shown that the CFL thickness depends on two parameters: the flow rate and the particle concentration. Accordingly, the CFL thickness increase by either: (i) lowering the PDMS concentration under equal flow rate, or (ii) increasing the flow rate for solutions of equal PDMS concentration, see Figure 11d. These tendencies are in agreemenst with those obtained for quasi-rigid particles [54,55]. RBC particulate fluid [54] showed higher CFL thickness than PDMS particulate fluid [52]. Alternatively, instead of PDMS microparticles, Lima et al. [59] investigated the CFL formation using flexible micelles of 1 wt% Brij L4 at the flow rate of 5 L/min, downstream of a microchannel with an abrupt contraction. The results obtained about the CFL thickness suggest a good agreement between both the proposed blood analogue and in vitro blood.

Nonetheless, a deeper study of the CFL phenomenon through different types of microchannels (geometries) should be done, comparing quantitatively (with higher accuracy) the results with that from real blood along the microchannels (both upstream and downstream of a contraction, bifurcation, etc.), to clarify the capacity of a blood particulate analogue fluid to replicate this phenomenon in in vitro studies.

Table 2 summarizes the information presented in Section 3.

## 4. Conclusions

In this review paper, a large variety of studies was shown providing important insights into how RBC templates of various materials can be engineered. Despite the promise of different production methods, several limitations must be overcome, such as low production rates, complexity of the fabrication, insufficient monodispersity, among others. All these limitations make the mass production of a blood particulate analogue fluid with haematocrit similar to real blood difficult. Flexibility of RBCs has been demonstrated to influence their mobility and biofunctions. Although this key property has been resembled approximately by several authors, further studies are needed to elucidate more details about this ability in most cases. PEG and PDMS particles, among others, demonstrated tunable elasticity, and they can be made to behave similar to RBCs under physiologically relevant conditions in a microfluidic blood capillary model. Although particles with RBC-like morphology may behave differently from their spherical counterparts concerning their physical performances (attachment, adhesion performance), the spherical PDMS particles showed in this review (by using a suitable mixing ratio) could be simple templates to mimic RBCs in blood particulate analogue fluids, overcoming all the limitations previously mentioned.

The majority of blood particulate analogue fluids are a viscoelastic fluid mixture developed to reproduce a reliable rheological functionality and flow phenomenon, which should closely match the entire characteristic behaviour of real blood. Thus, to conclude whether or not the developed blood particulate analogue fluid under the same in vitro flow conditions has similar reliable measurement to real blood, it is essential to investigate both its rheological characteristics and its CFL flow phenomenon (flow through complex microchannels). Most of the rheological measuring techniques usually have included the use of blood particulate analogue fluids under steady shear flow, while very limited works have been performed under oscillatory shear flow and extensional flow. The steady shear flow was conducted to characterize the solution viscosity compared to real blood, while, for a more complete understanding of the elastic contribution, the oscillatory shear, the SAOS flow is precisely used to measure the storage and loss modulus. In addition to the previous two techniques, the extensional flow measurement is used to measure the extensional relaxation time under the condition of pure uniaxial extensional flow, which was not possible under shear flow conditions. In summary, steady shear flow, as well as the oscillatory shear flow, and extensional flow measurement should not be ignored in future studies to evaluate whether or not the proposed analog fluid has viscous and elastic behaviour similar to real blood. The CFL is another important factor used to characterize the behaviour of proposed blood particulate analogue fluids. Its thickness analysis in in vitro studies should suggest a CFL thickness similar to in vitro real blood. As reported in the literature, the CFL thickness depends on several parameters, including the microchannel geometrical configuration, flow rate, and particle concentration. Further studies should be done to be able to obtain a particulate fluid (around 45% of particles) whose rheology and CFL phenomenon totally match with those of whole human blood.

Although there have been many advances, the development of a reliable blood particulate analogue fluid, with around 45% by volume of microparticles, continues to be a big challenge. The interest and applications of a reliable blood particulate analogue fluid are increasing over time. For instance, this desired dispersion could also be used as artificial blood to assess the flow and potential of microrobots for biomedical applications [104,105], in order to optimize organ-on-a-chip platforms for preclinical validation of advanced nanomedicines [106] and to study cardiovascular diseases in in vitro micro and macro-scale arterial stenotic biomodels [107].

## Figures and Tables

**Table 1:** Summary of RBC templates used in the literature.

Material	Shape	Size	*E* (Deformability)	Biological Functionality	Production Method	Production Rate	References
PLGA	Biconcave discoidal	7 ± 2 μm (diameter), around 2 μm (thickness)	*E* = 92.8 ± 42 kPa (high)	Yes	Electrohydrodynamic jetting	1 g/h	[34]
PEG (hydrogel)	Discoidal	8 ± 0.2 μm (diameter), 2 ± 0.1 μm (thickness)	(High)	No, but possible	Stop flow lithography	0.01 g/h	[35]
HEA (hydrogel)	Discoidal	5.2–5.9 μm (diameter), 1.22–1.54 μm (thickness)	*E*≥ 7.8 kPa (high)	No, but possible	PRINT^®^	-	[37]
PES	Spherical, slightly RBC-shaped	10 μm	*E* ≈ 2.6 GPa (low)	No	Electrospraying (electrohydrodynamic)	-	[39]
TEGA (hydrogel)	Discoidal	6.3 μm (diameter) × 1.8 μm (thickness)	*E*≥ 6.5 kPa (high)	Yes	PRINT^®^	-	[38]
PAH+GA	Biconcave discoidal (hollow)	6.7 μm (diameter) × 2.8 μm (thickness)	*E*(capsule wall) ≥ 100 MPa (high)	Yes	Layer-by-Layer from solid template	-	[41]
PEG (hydrogel)	Spherical	7–9 μm	0.2 ≤*E*≤ 3.3 kPa (high)	No, but possible	Layer-by-Layer from porous template (also called mesoporous silica templating method)	-	[42]
HA	Spherical (hollow)	7 μm	8.3 ≤*E*≤ 24.5 kPa (high)	No, but possible	$CAP_{ATRP}$ from solid template	-	[43]
HA	Spherical	6–8 μm	4.3 ≤*E*≤ 38.3 kPa (high)	No, but possible	$CAP_{ATRP}$ from porous template	-	[43]
PMMA	Spherical	6.32 ± 0.118 μm	3 ≤*E*≤ 3.3 GPa (low)	No	Unknown (Spheromers^®^ CA 6, Microbeads AS)	-	[50,54]
PMMA	Spherical	10 μm	3 ≤*E*≤ 3.3 GPa (low)	No	Unknown (Spheromers^®^ CA 10, Microbeads AS)	-	[55]
PDMS (6:4)	Spherical	7.13 ± 1.34 μm	*E* ≈ 1300 kPa (high)	No, but possible	Liquid-liquid flow-focusing with a hypodermic needle	0.01 g/h	[44,45,46]
Chitosan	Concave	7.4 ± 0.74 μm	*E* ≈ 9 MPa (low)	No, but possible	Electrospray with solvent diffusion	0.01 g/h	[47]
GUV (lipid)	Spherical	6.15 ± 1.24 μm	(High)	No	Lipid film hydration	0.01 g/h	[49]
PDMS (30:1)	Spherical	9.05 ± 2.5 μm	*E* ≈ 90 kPa (high)	No, but possible	Two syringe membrane emulsification	1 g/h	[50]
PS	Spherical	11.1 ± 0.208 μm	3 ≤*E*≤ 3.3 GPa (low)	No	Unknown (Dynoseeds TS10 Microbeads^®^ AS)	-	[50]
PLGA	Spherical	9.23 ± 0.35 μm	*E* ≈ 4300 MPa (low)	No, but possible	512-channel geometric droplet-splitting microfluidic device combined with a post array part	1 g/h	[51]
Brij L4 surfactant (micelles)	Spherical	7.72 ± 3.72 μm	(High)	No	Premix membrane emulsification	1 g/h	[59]
PDMS (6:4)	Spherical	7.1 ± 1.6 μm	*E* ≈ 1300 kPa (high)	No, but possible	Premix membrane emulsification	1 g/h	[52]

**Table 2:** Summary of blood particulate analogue fluids analyzed in the literature, including the rheological measuring techniques.

References	RBC Template Used	Liquid as Plasma	Particle Concentration	Shear Rheology (at Around 22 °C)	Extensional Rheology	Other Measurements
[29]	Quasi-rigid PS (around 1 μm)	Distilled water; distilled water + ${\mathrm{CaCl}}_{2}$ at 0, 10, 20, and 30 mM; Dx70 + ${\mathrm{CaCl}}_{2}$ at 10 mM	12, 24, and 32 wt%	Steady shear flow 0 ≤$\dot{\gamma }$ (s${}^{-1}$) ≤ 120; Oscillatory shear flow 0.02 ≤ω (rad/s) ≤ 0.8	No	Rouleaux (aggregation)
[38]	TEGA (hydrogel) with Hb (around 6 μm)	PBS	40 vol%	Steady shear flow 0.1 ≤$\dot{\gamma }$ (s${}^{-1}$) ≤ 10${}^{4}$	No	No
[54]	Quasi-rigid PMMA (around 6 μm)	Dx40 + SDS; Dx40 + XG (115 ppm) + SDS	5 wt%	Steady shear flow 1 ≤$\dot{\gamma }$ (s${}^{-1}$) ≤ 10${}^{4}$	No	CFL, deformability, and Rouleaux (aggregation)
[44]	Flexible PDMS 6:4 (around 6 μm)	Dx40	1 vol%	Steady shear flow 1 ≤$\dot{\gamma }$ (s${}^{-1}$) ≤ 10${}^{4}$	No	No
[55]	Quasi-rigid PMMA (around 10 μm)	Dx40; Dx40 + XG (115 ppm)	5 and 20 wt%	Steady shear flow 1 ≤$\dot{\gamma }$ (s${}^{-1}$) ≤ 10${}^{4}$; SAOS 0.01 ≤ ω (rad/s) ≤ 100; LAOS at ω = 0.1 and 1 (rad/s)	No	CFL, deformability, and Rouleaux (aggregation)
[49]	Flexible GUV (around 6 μm)	Tris-HCl buffer solution	1.6, 2.1 and 2.5 vol%	Steady shear flow 10 ≤$\dot{\gamma }$ (s${}^{-1}$) ≤ 10${}^{4}$	No	Deformability
[46]	Flexible PDMS 10:1, 8:2, 6:4, black 1:1, and red-dyed 10:1 (around 8 μm)	Dx40	8 vol%	Steady shear flow 1 ≤$\dot{\gamma }$ (s${}^{-1}$) ≤ 10${}^{4}$	No	CFL and deformability
[59]	Flexible micelles of Brij L4 surfactant (around 8 μm)	Pure water	1, 5, 10, and 20 wt%	Steady shear flow 1 ≤$\dot{\gamma }$ (s${}^{-1}$) ≤ 10${}^{4}$	No	CFL and deformability
[52]	Flexible PDMS 6:4 (around 7 μm)	Aqueous solution of 4 wt% SDS	8, 17, 21, 24, and 32 wt%	Steady shear flow 1 ≤$\dot{\gamma }$ (s${}^{-1}$) ≤ 3 × 10${}^{3}$; LAOS at ω = 0.158 and 1 (rad/s)	Yes	CFL and deformability
